# Pathophysiological classification of chronic rhinosinusitis

**DOI:** 10.1186/1465-9921-6-149

**Published:** 2005-12-19

**Authors:** James N Baraniuk, Hilda Maibach

**Affiliations:** 1 Georgetown University Proteomics Laboratory, Division of Rheumatology Immunology and Allergy, Room B105, Lower Level Kober-Cogan Building, Georgetown University, 3800 Reservoir Road, NW Washington, DC 20007-2197, USA

## Abstract

**Background:**

Recent consensus statements demonstrate the breadth of the chronic rhinosinusitis (CRS) differential diagnosis. However, the classification and mechanisms of different CRS phenotypes remains problematic.

**Method:**

Statistical patterns of subjective and objective findings were assessed by retrospective chart review.

**Results:**

CRS patients were readily divided into those with (50/99) and without (49/99) polyposis. Aspirin sensitivity was limited to 17/50 polyp subjects. They had peripheral blood eosinophilia and small airways obstruction. Allergy skin tests were positive in 71% of the remaining polyp subjects. IgE was<10 IU/ml in 8/38 polyp and 20/45 nonpolyp subjects (p = 0.015, Fisher's Exact test). CT scans of the CRS without polyp group showed sinus mucosal thickening (probable glandular hypertrophy) in 28/49, and nasal osteomeatal disease in 21/49. Immunoglobulin isotype deficiencies were more prevalent in nonpolyp than polyp subjects (p < 0.05).

**Conclusion:**

CRS subjects were retrospectively classified in to 4 categories using the algorithm of (1) polyp vs. nonpolyp disease, (2) aspirin sensitivity in polyposis, and (3) sinus mucosal thickening vs. nasal osteomeatal disease (CT scan extent of disease) for nonpolypoid subjects. We propose that the pathogenic mechanisms responsible for polyposis, aspirin sensitivity, humoral immunodeficiency, glandular hypertrophy, eosinophilia and atopy are primary mechanisms underlying these CRS phenotypes. The influence of microbial disease and other factors remain to be examined in this framework. We predict that future clinical studies and treatment decisions will be more logical when these interactive disease mechanisms are used to stratify CRS patients.

## Introduction

The syndrome of chronic rhinosinusitis (CRS) has been defined by mucopurulent anterior or posterior nasal discharge, regional facial or dental pain, sinus region tenderness, fetid odor, and other symptoms that do not respond to 12 weeks of adequate therapy [1,2]. This clinical definition has been updated to divide CRS into those with ("CRSwNP") and without nasal polyposis ("CRSsNP"; "s" = without) [3-5]. However, additional differences in presentation, natural history, background of atopy or other phenotypes, eosinophilia, pathophysiological mechanisms, and responses to therapy may occur within each subset. A classification based on pathophysiological mechanisms would be valuable for stratifying patients for optimal treatment and for clinical study [5-8].

The complexity of CRS is apparent from the many individual risk factors that have been associated with this diagnosis, and the inability of any single risk factor to explain the syndrome. Factors include atopy, humoral immunodeficiency and other immune deviations, autocrine and paracrine eosinophilic disease, aspirin and other nonsteroidal antiflammatory drug (NSAID) sensitivity ("Triad Asthma"), nasal polyposis, and glandular hypertrophy [7,8]. Many reductionist studies have investigated individual aspects of CRS, but these were generally not designed to simultaneously examine multiple clinical and objective variables that may discriminate between phenotypes.

Because of the wide spectrum of opinions in the literature, we chose to return to "first principles" and evaluate, rank and classify subjects into logical subsets of CRS pathology. We hypothesized that the analysis of multiple variables in well characterized CRS subjects would lead to a better understanding of the relationships between variables. These insights may generate new hypotheses to explain the discrete histopathological subsets of CRS [1-8].

This first pilot study was a retrospective analysis of the last 100 consecutive chronic sinusitis subjects seen by one allergist in a tertiary care setting. Limitations due to potentially biased patient referral patterns and examination of more severe patients than commonly seen in general practice were recognized at the onset. However, retrospective analysis was required to define the most critical factors associated with CRS so that prospective studies could focus on the most relevant issues. Variables included demographics, aspirin – NSAID sensitivity, allergy skin test results, pulmonary function tests, serum IgE and other immunoglobulin (Ig) subclass levels, and peripheral blood eosinophilia. Data were collated and variables converted to qualitative measures to facilitate contingency table (Chi^2^) analysis. This identified the most prevalent variables, and permitted logical subdivision of the study population. The aim was to identify the most coherent algorithm for clinical evaluation of CRS subjects.

The study population was split into groups with nasal polyps, and the remainder who did not have nasal polyps [3-5]. The polyposis group was subdivided by the presence of aspirin sensitivity into those with **n**asal **p**olyps and **aspirin **sensitivity (**NPasa**), and **n**asal **p**olyps with other features (**NPother**). Subjects without polyps were subdivided based on CT scan evidence of nasal disease only, or nasal + sinus mucosal thickening > 5 mm. The group with only narrowing of the **o**steo**m**eatal **c**omplex (**OMC**) was separated from subjects with sinus involvement (CRSsNP). This represents a modification of consensus guidelines [4,5] by limiting the **CRSsNP **group to those with radiological evidence of sinus involvement. In the absence of nasal polyposis, we proposed that the sinus thickening in the CRSsNP group was due to glandular hypertrophy [7,8].

Portions of this work have been presented as abstracts at scientific meetings [9,10].

## Methods

### Subjects

Charts from 100 consecutive chronic sinusitis subjects were assessed retrospectively. The clinical diagnosis of chronic sinusitis was made by contemporary criteria [1,2] based on chronic nasal discharge, sinus region pain and tenderness, and poor symptomatic responses to antibiotics and other therapies for at least 12 weeks. Most gave a history of recurrent acute sinusitis that progressed to CRS over a period of several years. Patients were referred by otolaryngologists, pulmonologists, general internists, and by self-referral. Subjects with allergic rhinitis or nonallergic rhinitis without chronic sinusitis complaints were excluded.

Independent groups of CRS, allergic rhinitis, and healthy subjects with neither condition provided representative control groups. They were recruited to concurrent clinical research studies of fatigue, pain sensitivity, irritant rhinitis, and tobacco sensitivity that did not include CRS as an inclusion or exclusion criterion [11,13-18]. However, because of the nature of their studies, they did not have the same extensive laboratory evaluation at the clinical CRS patients.

### Variables

CT scan severity was used as a study variable and so was not required for the clinical diagnosis of sinusitis [1,2]. Coronal CT scans were scored according to the May classification [20] in order to be consistent with our previous studies [7,8]. Normal nasal and sinus CT scans were scored as Grade 0. Grade 1 indicated osteomeatal narrowing without sinus mucosal thickening (OMC). Thickening or opacification limited to the ethmoid sinuses was Grade 2 disease. Grade 3 required bilateral disease involving mucosal thickening, air-fluid levels, or opacification of individual larger sinuses. Pansinusitis with opacification of ethmoid, maxillary, frontal and potentially sphenoid sinuses was classified as Grade 4. In practical terms, 3 groups were identified. The OMC group had nasal disease only (Grade 1). Sinus involvement (Grades 2 to 4) was present in both the polyp and CRSsNP groups.

Other variables included age; gender; race and ethnicity; strong and convincing history of aspirin or NSAID sensitivity causing airway or angioedema symptoms; the presence of polyps by visual, rhinoscopic, or surgical examination; blood eosinophilia; serum immunoglobulin (Ig) concentrations; pulmonary function tests; and allergy skin test results. The highest eosinophil counts and most deleterious pulmonary function and Ig results were recorded in order to emphasize distinctions between subjects. Eosinophil counts over 4% were scored as elevated (score = 1 vs. ≤ 4% = normal; score = 0). The mean value was determined for all subjects with counts >4%.

The serum concentrations of IgE, IgA, IgM, IgG1, IgG2, IgG3, and IgG4 were measured at 3 clinical laboratories. Unfortunately, over the time period of this study, the ranges of normal for each isotype changed in each laboratory. This may have reflected each laboratory's individual efforts to define normal ranges. As a result, we qualitatively defined Ig isotype levels as either "normal" or below the lower limits of normal for each laboratory ("deficient"). Since there is no absolute lower limit of normal for IgE, these concentrations were converted to a qualitative, logarithmically-based scale with levels of <0 IU/ml ("absent IgE") [19], 10 to 99.9 ("normal"), and > 100 IU/ml ("elevated"). The independent control subjects had IgE measured in parallel using the same laboratories [13,14]. The other immunoglobulin isotypes were measured and qualitatively scored as normal or elevated (score = 1), or below the lower limit of normal (score = 0) for the specific laboratory doing the test.

Puncture skin tests to geographically significant allergens were scored on a 0 to 4 point scale as previously described [11,12]. The allergens were birch, maple, oak, grass mix, rye grass, ragweed, plantain, cat, dog, cockroach, *Dermatophagoides farinae, D. pteronyssinus, Alternaria, Aspergillus, Epicoccum, Fusarium, Helminthosporium, Monilia*, and *Penicillium *(Hollister-Stier, Spokane, WA). If the histamine was 2+ or less and no allergen test was > 3+, then intradermal tests were performed with mixed trees, Southern grass mix #5, ragweed, mixed weeds, cat, dog, cockroach, the 2 dust mites, and mixed molds. If 2 or more tests had results at the 3+ or 4+ levels, then the subject was considered "skin test positive". Both the quantitative number of positive skin tests, and the qualitative, nominal "positive" (score = 1) and "negative" (score = 0) results were recorded. Data were then tabulated for trees, grasses, weeds, ragweed, fungi, cat (included all dog sensitive subjects), *D. farinae *and *D. pteronyssinus *(included all cockroach reactors).

Spirometry was recorded as the FEV1/FVC ratio, and absolute and percent of predicted values for FVC, FEV1, and FEF_25%–75%_. Percent predicted values were qualitatively scored as positive (score = 1) when < 70%, and normal (negative, 0) when ≥ 70%.

### Data analysis

All subject data were hand entered without patient identifiers into Excel (Microsoft, Redmond, WA) spreadsheets, and assigned random, anonymous 5 digit identification codes. No patient identifiers were included on the worksheets, and the codes were not recorded in patient charts. The data were visually inspected and verified. One subject did not meet the review criteria at this stage and was removed from consideration. Some CRS subjects did not have data for all the variables, but were retained in the database. The issue of missing data points was addressed in subset analysis by including only those subjects with the pertinent data. The data were transferred into a SAS 9.0 (Carey, NC) database for a further review of internal consistency and statistical analysis.

The frequency of each variable was determined for the study population. Frequencies in females and males were compared to assess gender effects. Continuous variables such as the number of positive skin tests and pulmonary function test results were compared between the NPasa, NPother, CRSsNP and OMC categories by ANOVA followed by 2-tailed, unpaired Student's t-tests. Bonferroni corrections for multiple comparisons were not used for this pilot investigation. Means or geometric means and 95% confidence intervals were displayed with significance defined for p < 0.05. Qualitative data (0, 1) such as the presence or absence of reduced airflow (e.g. FEV1/FVC ≤ 70% of predicted) or the presence of atopy were compared between these 4 categories by Fisher's Exact test between groups. The tables displayed these significance levels using a standard format for footnotes. Significant ANOVA results for the 4 groups were identified by superscript capital letters. Fisher's Exact test results were given in [] when proportions were compared to NPasa data, and {} when compared to NPother. T-test results were shown as footnotes for NPasa vs. the other 3 groups, OMC vs. NPother and CRSsNP, and NPother vs. CRSsNP.

Multivariate and principal component analyses were used to determine the variables that best characterized each group of patients. Factor analysis permitted inferences about potential common mechanisms within each category. Multilogistic and multilinear regression analysis were also applied, but the complexity of the interactions between variables did not define any significant, predictive models (e.g. general linear modeling).

## Results

### Demographics

The average age of the study population (n = 99) was 45.1 yr (42 to 47; mean and 95% C.I.) with 27% males. The racial composition was 88% Caucasian, 8% African-American, 3% Asian, and 5% Hispanic ethnicity. Drugs used by the 99 subjects were topical nasal glucocorticoids (n = 79), antihistamines (72), daily nasal saline irrigation (52), inhaled glucocorticoids (51), short- and long-acting bronchodilators (48), ipratropium bromide nasal spray (39), and leukotriene receptor antagonists (32).

### Stratification by physical examination, CT scan and presumed histology

As described in the introduction, CRS subjects were readily subdivided based on (a) polyposis (50% prevalence), (b) aspirin sensitivity (17% prevalence), and (c) the May grade of sinus CT scan severity (Grades 2, 3 and 4 versus Grade 1) (Table 1). The 1^st ^decision level was the presence (50/99) or absence (49/99) of polyps. Aspirin sensitivity was the 2^nd ^decision level, and was present in 34% of polyp but only 4% of nonpolyp subjects (p = 0.0001, Chi^2^). The polyp group was divided into those with aspirin/nonsteroidal anti-inflammatory drug sensitivity (NPasa; n = 17) and those without this sensitivity (NPother; n = 33). All the NPasa subjects had severe asthma or laryngospasm symptoms upon NSAID exposure. Two nonpolyp subjects had aspirin sensitivity, but their reactions were limited to urticaria and angioedema. They had no airway symptoms.

**Table 1:** Clinical subdivisions of chronic rhinosinusitis based on nasal polyposis and aspirin – sensitivity (mean with 95% CI, or % of group).

1^st ^Decision	**Chronic Rhinosinusitis (CRS; n = 99)**			
Nasal Polyps	**Present: N = 50**		**Absent: N = 49**	

2^nd ^Decision	Nasal Polyps with Aspirin Sensitivity (NPasa)	Nasal Polyps with Other Features (NPother)	CRS without (s) Nasal Polyps (CRSsNP)	Osteomeatal Complex Disease (OMC)
Aspirin Sensitivity	17/17 (100%) (airways)	0/33 (0%) [<10^-9^] (airways)	1/28 (4%) [<10^-9^] (urticaria)	1/21 (5%) [<10^-9^] (urticaria)
May CT Scan Grade ^A^	3.35 (3.02 to 3.69)	3.10 (2.85 to 3.34) ^¶¶^	2.43 (2.22 to 2.64) ^§§§ † ¶^	1 (1 to 1) ^§§§^
Blood Eos > 4% ^B^	11/17 (65%)	13/33 (39%)	9/27 (33%) [0.03]	4/20 (20%) [0.006]
% Males	4/17 (24%)	12/33 (36%)	8/28 (29%)	3/21 (14%)
Age (yr) ^C^	53.0 (48.1 to 57.9)	43.7 (39.1 to 48.3) ^§^	40.5 (35.7 to 45.4) ^§§^	46.7 (39.4 to 54.1)

ANOVA: ^A ^= 10^-20^; ^B ^= 0.042; ^C ^= 0.031. [p] = Fisher's Exact test vs. NPasa. Two-tailed, unpaired Student's t-tests: ^§ ^p = 0.02; ^§§ ^p = 0.002 and ^§§§ ^p < 2 × 10^-5 ^vs. NPasa; ^¶ ^p = 0.005 and ^¶¶ ^p < 10^-13 ^vs. OMC; ^† ^p = 0.005 vs. NPother.

The NPasa and NPother subjects had May CT scan Grades of 2, 3 and 4. The nonpolyp subjects were divided into 21 subjects with May Grade 1 (OMC, nasal disease only), and 28 subjects with May Grades 2, 3 and 4 (CRSsNP). Since Malekzadeh has demonstrated that polyp and glandular hypertrophy subsets were mutually exclusive [7,8], the CRSsNP subjects were assumed to have glandular hypertrophy. May CT scan severity grades were significantly higher for NPasa (3.35) and NPother (3.00) than CRSsNP (2.43) and OMC (1) groups (table 1). The NPasa group was significantly older than the NPother and CRSsNP groups.

Peripheral eosinophilia > 4% was qualitatively present in 65% of NPasa subjects. This was significantly higher than the CRSsNP (33%) and OMC (20%) (p < 0.01 for each comparison). Peripheral eosinophilia > 4% was intermediate in the NPother group (39%). When eosinophils were elevated, their mean concentration was 10.9% (8.9 to 12.9; n = 34 total).

### Stratification by spirometry

Asthma was highly prevalent in CRS (range 68% to 88%, table 2). "Triad Asthma" was present in 15/17 NPasa subjects. The qualitative finding of FEV_1 _/FVC ratios < 70% was present in 75% of the NPasa group. This group had significantly worse airflow obstruction than the NPother (41%), CRSsNP (21%) and OMC (14%) groups (p = 0.004 by ANOVA). FEF_25%–75% _was below < 70% of predicted in 91% of NPasa, compared to 55% of NPother, 29% of CRSsNP, and 43% of OMC (p = 0.014 by ANOVA).

**Table 2:** Asthma and spirometry in chronic rhinosinusitis subsets (mean, 95% CI; or percentage).

	Nasal Polyps with Aspirin Sensitivity (NPasa)	Nasal Polyps with Other Features (NPother)	CRS without Nasal Polyps (CRSsNP)	Osteomeatal Complex Disease (OMC)
Clinical Asthma	15/17 (88%) Triad Asthma	23/32 (72%)	19/28 (68%)	14/20 (70%)
Spirometry	N = 12	N = 22	N = 14	N = 14
FEV_1_/FVC (%) ^A^	64.4% (59.8 to 69.0)	70.9% (65.3 to 76.5)	78.0% (72.3 to 83.6) ^§^	79.8% (73.6 to 86.0) ^§§^
FEV_1_/FVC<70% ^A^	9/12 (83%)	9/22 (41%) [<0.05]	3/14 (21%) [0.008]	2/14 (14%) [0.003]
FEF_25%–75% _(%)	48.4% (35.9 to 60.8)	59.7% (47.9 to 71.5)	73.9% (58.0 to 89.7)	69.5% (55.8 to 83.2)
FEF_25%–75%_<70% ^B^	10/11 (91%)	11/20 (55%) [0.04]	4/14 (29%) [0.002]	6/14 (43%) [0.02]

ANOVA: ^A ^= 0.005; ^B ^= 0.014. [p] = Fisher's Exact test vs. NPasa. Two-tailed, unpaired Student's t-tests: ^§ ^p = 0.002, and ^§§ ^p = 0.0009 vs. NPasa.

### Stratification by positive allergy skin test results

The separate set of healthy control subjects had a frequency of positive allergy skin tests of 42.9% (41.9 to 43.9 n = 792). This "background rate" of positive results was compared to the CRS categories.

Skin tests were positive in 53 of 92 subjects (58%) (table 3). The remainder refused skin testing or had RAST tests. The latter were not used to determine atopy status because of variations between clinical laboratories over time regarding grading and the levels for positive results.

**Table 3:** Numbers of subjects per group with positive allergy skin tests (%).

	Nasal Polyps with Aspirin Sensitivity (NPasa)	Nasal Polyps with Other Features (NPother)	CRS without (s) Nasal Polyps (CRSsNP)	Osteomeatal Complex Disease (OMC)
Group Sizes (N)	16	31	28	17
N (%) Positive	7 (44%)	22 (71%) {0.03}	19 (68%)	7 (41%)
Trees	5 (31%)	12 (39%) {0.04}	10 (36%)	2 (12%)
Grasses	6 (38%)	10 (32%)	10 (36%)	4 (24%)
Ragweed	2 (13%)	11 (36%)	8 (29%)	2 (12%)
Weeds	4 (25%)	5 (16%)	7 (25%)	2 (12%)
Mites (*Df, Dp*)	6 (38%)	15 (48%)	14 (50%)	5 (29%)
Cat	4 (25%)	13 (42%)	9 (32%)	3 (18%)
Dog	3 (19%)	6 (20%)	4 (14%)	0 (0%)
Fungi	3 (19%)	6 (20%)	10 (36%)	4 (24%)
Cockroach	1 (6%)	4 (13%)	5 (18%)	2 (12%)
Persistent ^§^	6 (38%)	21 (68%) [0.04]	17 (61%)	7 (41%)
Subjects with IgE <10 IU/ml but positive skin tests	#1. Trees, grasses, weeds, *Df, Dp*, cat	#1. Grasses	#1. Trees, *Df, Dp* #2. Cat #3. Trees, grasses	#1. Grasses, ragweed, weed, fungi, cat

[p] = Fisher's Exact test vs. NPasa; {p} vs. OMC. *Df, Dermatophagoides farinae; Dp*, *D. pternonyssinus*; ^§ ^Persistent was defined as at least 1 positive result to fungi, cat, *D. farinae *or *D. pteronyssinus*.

The control level of 43% was the same as for NPasa (44%) and OMC (41%). This suggested that atopy was present in each category, but may have been a coincidental co-morbidity. Allergic rhinitis may have been present, but was unlikely to be a primary mechanism of CRS pathogenesis in these two categories. Instead, other nonallergic mechanisms must have predominated.

Positive skin tests were more common in the NPother (71%) and CRSsNP (68%) groups. The proportion of excess cases associated with atopy was 28% for NPother (71% minus 43%) and 25% (68% minus 43%) for CRSsNP. Atopy may have had a more significant pathogenic role in these two categories by modifying or exacerbating other mechanisms responsible for polyposis and glandular hypertrophy.

Dust mites, cat, trees, ragweed and grasses were the groups of allergens with the highest frequencies of positive results in the NPother and CRSsNP categories (table 3). Overall, 55% of CRS subjects had responses to "persistent" dust mite, cat, and fungal allergens. Only 4% had solely seasonal allergen reactivity. A clinical relationship was noted between autumn (ragweed) and persistent (perennial, dust mites, cat, fungi) allergen sensitization, viral upper respiratory tract infections, and exacerbations of chronic sinusitis that peaked between October and December in our locale (personal observation).

Curiously, 6 subjects with IgE < 10 IU/ml had positive allergy skin tests (bottom line, table 3). Three were in the CRSsNP group. We speculate that these represented persons who had lost the ability to synthesize substantial amounts of circulating IgE, but still had allergen-specific IgE bound to their cutaneous mast cells. This may indicate a dynamic collapse of IgE production or B cell function in hypertrophic chronic sinusitis (CRSsNP). Two of these subjects had late phase responses indicating maintenance of allergen-specific Th2 lymphocyte reactivity.

Eosinophil counts and the logarithm of IgE concentrations were assessed. They were positively correlated only for those subjects with negative allergy skin tests (ρ = 0.46; p < 0.05). Peripheral blood eosinophilia was independent of skin test reactivity. This suggested that unknown nonallergic mechanism(s) contributed to both eosinophilia and higher IgE levels in CRS.

### Stratification by immunoglobulin deficiencies

About two-thirds of the population had measurements of immunoglobulin isotypes including IgG subclasses. The proportions of subjects per category with isotype levels below the lower limits of normal and/or IgE < 10 IU/ml were shown in table 4. The median number of low isotypes per subject was 1.5 in NPasa, 0.5 in NPother, 2.5 in CRSsNP and 1.0 in OMC. Strikingly, IgE was low in 44% of CRSsNP and 45% of OMC subjects. These proportions were significantly higher than NPother (17%; p = 0.03 by Fisher's Exact tests). NPasa had an intermediate frequency and lower sample size, and so was not significantly different. Low serum IgE was most frequent in nonpolypoid CRS groups.

**Table 4:** Frequencies of immunoglobulin isotypes below the lower limits of normal.

	Nasal Polyps with Aspirin Sensitivity (NPasa)	Nasal Polyps with Other Features (NPother)	CRS without (s) Nasal Polyps (CRSsNP)	Osteomeatal Complex Disease (OMC)
IgE<10 IU/ml	4/14 (29%)	4/24 (17%)	11/25 (44%) {0.03}	9/20 (45%) {0.03}
IgA	2/12 (17%)	2/21 (10%)	5/18 (28%)	2/14 (14%)
IgM	1/12 (8%)	3/21 (14%)	7/18 (39%)	3/14 (21%)
IgG1	4/12 (33%)	5/21 (24%)	8/18 (44%)	5/14 (36%)
IgG2	2/12 (17%)	2/21 (10%)	4/18 (22%)	2/14 (14%)
IgG3	3/12 (25%)	3/21 (14%)	8/18 (44%)	3/14 (21%)
IgG4	1/12 (8%)	3/21 (14%)	5/18 (28%)	3/14 (21%)
IgE+IgG1/3 * ^A^	2/12 (17%)	1/19 (5%)	8/18 (44%) {0.007}	2/14 (14%)

* IgE <10 IU/ml plus either low IgG1 or IgG3. ANOVA: ^A ^= 0.02. {p} = Fisher's Exact test vs. NPother.

**Figure 1:**
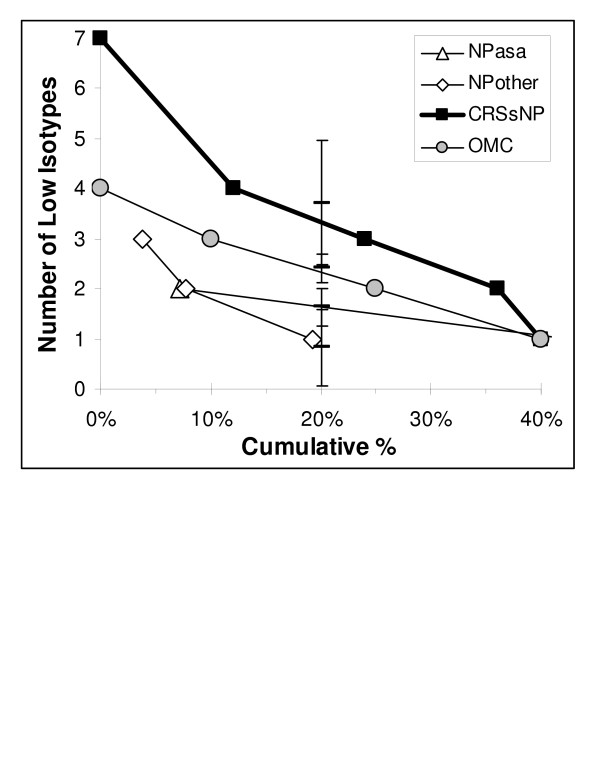
The number of low isotypes was plotted against the proportion of each group having these deficiencies. Low isotypes (n = 7) were identified in 3 CRSsNP and 1 NPother subject. Fewer nasal polyp subjects had low isotypes compared to the nonpolypoid pair of groups. This was demonstrated by the 95% confidence intervals at the midpoint of these curves (bars with error bars at 20%). Polyp and nonpolyp confidence intervals did not overlap. Most of the subjects had no humoral immune deficits (zero low isotypes, not depicted).

Immunoglobulin subclass deficiencies were more frequent in CRSsNP than NPother for IgG3 (44% vs. 14%), IgA (28% vs. 10%), and IgM (39% vs. 14%). Low IgM was more prevalent in CRSsNP than NPasa (39% vs. 8%). The small numbers of subjects per group precluded statistical significance. However, subjects with low IgE (<10 IU/ml) plus low levels of either IgG1 or IgG3 were found more frequently in the CRSsNP group (44%; p = 0.02 by ANOVA). It was surprising to find such a high proportion of CRS subjects with low levels of IgE and IgG subclasses compared to IgA deficiency (table 5) [19,21,22]. The numbers of subjects in each group with multiple isotypes below the lower limits of normal were assessed. Both the CRSsNP and OMC groups had higher proportions of subjects with several low isotypes compared to the NPasa and NPother groups (p < 0.05 for each comparison). This was demonstrated by plotting the proportion of each group who had low isotypes against the number of these deficiencies per individual (figure 1). Curves were compared at the midpoint of this range (20% cumulative proportion for each group). These humoral immune deficits may have played a permissive role in the development of the glandular hypertrophy that was presumed to occur in CRSsNP [7,8].

**Table 5:** Qualitative stratification of clinical disorders and positive allergy skin tests by serum IgE (geometric mean, 95% C.I.).

	**IgE < 10 IU/ml**	**10 ≤ IgE ≤ 100 IU/ml**	**IgE > 100 IU/ml**
IgE	1.7 (0.8 to 2.9)	35.1 (28.1 to 43.7)	277 (212 to 364)
Clinical Asthma ^B^	14/29 (48%)	23/28 (82%) [0.005]	24/29 (83%) [0.005]
FEV1/FVC ≤ 70%	5/17 (29%)	7/18 (39%)	10/18 (56%)
FEF_25%–75% _≤ 70%	7/17 (41%)	11/17 (65%)	12/18 (67%)
Nasal Polyps	9/29 (31%)	15/28 (54%)	17/29 (59%) [0.02]
Eosinophilia > 4%	8/28 (29%)	12/28 (43%)	16/29 (55%) [0.03]

**Positive Allergy Skin Test Results**			

N per group	28	26	26
+ Results/subject **†**	0.74 (0.10 to 1.30)	3.5 (1.82 to 5.43) ^†^	5.08 (3.90 to 6.27) ^††^
Trees ^C^	3 (11%)	6 (23%) {0.01}	15 (58%) [0.0003]
Grasses ^A^	4 (14%)	8 (31%)	12 (46%) [0.009]
Weeds	2 (7%)	7 (27%) [0.047]	4 (15%)
Ragweed ^C^	0 (0%)	8 (31%) [0.002]	12 (46%) [0.00003]
Fungi ^B^	1 (4%)	5 (19%) {0.049}	11 (42%) [0.0006]
Cat ^B^	3 (11%)	7 (27%)	13 (50%) [0.0002]
*D. farinae *^D^	1 (4%)	12 (46%) [0.0002]	18 (69%) [10^-6^]
*D. pteronyssinus *^D^	2 (7%)	10 (39%) [0.006] {0.03}	17 (65%) [10^-5^]
"Persistent" ^§D^	4 (14%)	15 (58%) [0.001] {0.01}	23 (88%) [10^-8^]

ANOVA: ^A ^= 0.04; ^B ^= 0.004; ^C ^= 0.0003; ^D ^< 0.00003. [p] = Fisher's Exact test vs. IgE<10 IU/ml; {p} vs. IgE>100 IU/ml. **† **Number of positive allergy skin test results per subject (mean; 95% CI): ^† ^p = 0.003 and ^†† ^p = 10^-7 ^vs. IgE<10 IU/ml by 2-tailed, unpaired Student's t-test.

### Stratification by IgE concentrations

Subject results were stratified by the logarithmically transformed serum IgE levels into <10 (low), 10 to 100 (normal), and >100 IU/ml (elevated) subsets (table 5). Clinical asthma had half the prevalence in the low IgE group compared to the normal and elevated IgE groups (p = 0.004 by ANOVA). This suggested the presence of nonatopic asthma. Measures of airways obstruction, polyposis and peripheral eosinophilia were not significantly different between IgE subsets. As expected, the low IgE group had lower rates of positive allergy skin tests and fewer positive results per subject. Reactivity was highest in the high IgE group.

### Factor analysis of the entire population

Principal component analysis of the entire population was performed to determine if a second, independent statistical method would verify the results of the stratification process, and provide additional mechanistic insights. The initial analysis started with all variables, and could be forced to a final result of 2 factors: (i) polyposis, and (ii) positive allergy skin tests. Additional analyses were run to improve the efficiency and balance by eliminating co-variates (e.g. asthma and pulmonary function test results), redundant (individual qualitative assessments of reduced immunoglobulin isotype concentrations), and insignificant (age, gender and ethnicity) variables. The final analysis had optimal efficiency and balance between six factors (table 6).

**Table 6:** Factor analysis for the entire CRS population. The variables that predicted CRS in the most similar fashion were grouped together as Factors. Factors 1 (persistent dust mite) and 3 (seasonal pollens) implicated allergic rhinitis mechanisms. Factor 2 related asthma with polyposis. Independent factors were eosinophilia, aspirin sensitivity and low immunoglobulins.

Factors	Variables	Loading factors	Explained variance	Eigenvalue
Factor 1	Positive dust mite skin test Positive skin tests for persistent allergens (dust mites, cat, and fungi) Clinical diagnosis of allergic rhinitis	0.93 0.91 0.90	29%	3.5
Factor 2	FEV_1_/FVC ratio (continuous range) FEF_25%–75% _≤ 70% of predicted (score = 1) Polyposis	-0.93 0.92 0.77	26%	3.2
Factor 3	Positive skin test results to weeds Positive skin test results to grasses	0.91 0.79	15%	1.6
Factor 4	Blood eosinophils > 4% (qualitative)	0.92	12%	0.9
Factor 5	Aspirin sensitivity	0.90	10%	0.8
Factor 6	Any immunoglobulin < lower limits of normal	0.70	8%	0.6

The factors were consistent with the stratification process. Factor 1 represented active persistent rhinitis symptoms with positive allergy skin tests to indoor and year-round allergens. The rhinitis symptoms plus positive skin tests supported the diagnosis of allergic rhinitis. Seasonal allergies were represented by Factor 3. These results were consistent with the rate of atopy in this (59%, table 3) and the control populations (43%).

Factor 2 related large and small airways obstruction with nasal polyposis. The association of more severe asthma with polyposis implied an association of milder or no asthma in the nonpolypoid group. Factor 5 was aspirin sensitivity which justified the designation of an independent category of nasal polyposis (NPasa).

Factor 4 of peripheral blood eosinophilia > 4% was independent of other variables. This was understandable, since mechanisms of aspirin sensitivity, polyposis, asthma, and allergic rhinitis may all cause eosinophilia.

Factor 6 was the qualitative assessment that an individual had one or more immunoglobulin isotype below the normal range. More complete, quantitative immunoglobulin data may have generated stronger relationships given the frequencies of abnormal results in the nonpolypoid CRSsNP and OMC subjects (table 2).

### Factor analysis of asthma and atopy in each subgroup

Asthma and positive allergy skin test results were important defining variables in the preceding factor analysis. Additional factor analyses were performed for each of the CRS subgroups to better define potential mechanistic interactions. Measures of lung function, immunoglobulins and eosinophils were excluded to maintain the focus on patterns of allergy skin test results.

### NPasa

Clinical asthma was present in 15 of 17 NPasa subjects ("Triad Asthma"). This suggested that the nonallergic mechanism(s) of aspirin sensitivity was highly associated with the pathology of both the chronic sinusitis and asthma. These mechanisms could include autonomous eosinophilia, tissue remodeling by other resident cells, and glucocorticoid resistance. Factor analysis defined only one additional significant factor: older age. These defining features accounted for essentially all of the explained variance within the NPasa group. Atopy was not a defining factor for NPasa.

### NPother

Factor 1 was defined by positive allergy skin test results to cat, tree, grass, and ragweed (loading factor = 1.0 for each). Atopy may have contributed to, or exacerbated, nasal polyp formation, CRS, and/or asthma in the 23 skin test positive NPother subjects (n = 32; table 3). Factor 2 suggested an independent mechanism with older age (0.95), higher CT scan severity grades (0.85), positive skin tests to weeds (0.88) but negative loadings for dust mites (-0.88 for each; i.e. not sensitive to dust mites). A nonatopic mechanism was suggested by the negative loading factor for dust mites. The significance of the reactivity to weeds in this model was questionable since this set of allergens had the lowest frequency of positive skin tests. It would be of interest to determine if the nonatopic NPother subset defined by Factor 2 (9/32 subjects) had subclinical aspirin sensitivity.

### Nonpolypoid subjects

Positive skin test reactivity was evident in the nonpolypoid group. Factor 1 contained both of the dust mites. Factor 2 contained the seasonal pollens. Factor 3 was defined by cat and fungi. Factors 1 and 3 were components of the "persistent" allergen grouping.

### CRSsNP

Factor 1 encompassed positive skin tests to fungi and trees plus the absence of Chronic Fatigue Syndrome (explained variance = 27%). Factor 2 included weed and grass sensitivity (20%); Factor 3 dust mites (20%); and Factor 4 ragweed and cat (19%). The cumulative explained variance was 86% indicating the strong influence or co-variance of atopy in the CRSsNP group. The negative loading of Chronic Fatigue Syndrome was an important finding indicating that atopy, immunoglobulin dysfunction, polyposis and sinusitis (May grades 2, 3 or 4) were unlikely to be of pathological significance in this syndrome. Instead, mechanisms such as nociceptive hyperalgesia and allodynia were more likely to be responsible for "sinus" complaints in Chronic Fatigue Syndrome.

### OMC

The OMC group was similar to CRSsNP. Factor 1 incorporated weed, tree, cat, and fungal sensitivity (32%). Factor 2 was hypersensitivity to *D. farinae*, *D. pteronyssinus *and ragweed (27%). Factor 3 was distinct since it involved age (0.95) and FEV_1_/FVC (-0.93) (20%). Factor 3 related older age to worse airways obstruction.

## Discussion

### Limitations

This descriptive, observational study was limited by the amount of information that could be collected in a reliable manner. Surgical, intramaxillary sinus puncture, pathological (e.g. presence of allergic mucin), and microbial culture results were not available on a consistent basis. Smears, brushings or Rhinoprobe scrapings of the nasal mucosa, especially directed towards the osteomeatal complex were not routinely performed. Identification of significant nasal eosinophilia or neutrophilia would have added another inflammatory dimension to the analysis. Subjects with nonallergic rhinitis with eosinophilia syndrome (NARES) or with blood eosinophilia (BENARES) were not identified. Factor analysis demonstrated that fungal sensitivity and polypoid disease were not associated in this population. This was consistent with the low frequencies of clinical allergic fungal sinusitis and positive allergy skin tests to fungi in this unique set of patients (table 3).

Pulmonary function, peripheral blood immunoglobulin and eosinophil information were incomplete for the entire population. This was overcome by stating the numbers of subjects involved in each statistical comparison. The wide ranges for some of the data required stratification, logarithmic transformation, and qualitative analysis to identify significant trends.

### Stratification

The most informative stratification tactic was to divide CRS subjects into those with and without polyps as suggested by recent consensus statements [3,4]. Polyposis can be identified by direct visualization, rhinoscopy or at surgery. Polypoid changes may be inferred from CT scans unless the changes were early or copious mucus secretions obscured the outlines of polypoid masses. Early polypoid changes such a middle turbinate (May Grade 1) or ethmoid disease (May Grade 2) may require medial middle turbinate biopsy and histological examination for diagnosis [8,23,24].

The polypoid subjects were subdivided based on their sensitivity to aspirin and other nonsteroidal anti-inflammatory drugs. The histories of asthma or laryngospasm after taking one or more of these drugs were convincing. The pulmonary function tests and review of current medications confirmed the presence of reversible airflow obstruction in the NPasa subset. The prevalence of aspirin sensitivity in adult asthma was recently estimated at 21% (14% to 29%; 95% CI) [23]. Our results suggest that 17% of CRS and one third of all nasal polyp subjects have aspirin sensitivity. Nonpolyp subjects did not have aspirin – induced airway symptoms.

The overall rate of positive allergy skin tests was 59% in this population. Aspirin sensitivity with asthmatic or laryngeal symptoms were present in 7/55 skin test positive and 10/44 skin test negative subjects. Factor analysis demonstrated that aspirin sensitivity was not associated with any reproducible pattern of skin test responses. Positive allergy skin tests were present in only one third of the NPasa group, but in two thirds of the remainder of the nasal polyp (NPother) group. This suggested that mechanism(s) responsible for polyp formation predominated in NPasa and NPother, but that atopy modified the expression of CRS in the allergic NPother subset. We suggest that subclinical aspirin sensitivity may occur in NPother subjects with negative skin tests, and that aspirin provocations may be required for diagnosis [25]. The presence of aspirin sensitivity was not examined in previous studies that found allergic rhinitis in 84% of endoscopic sinus surgery patients [26], 54% of CRS outpatients [27], or 37% of children with sinusitis [28].

These findings raise the important question of what constitutes allergic rhinitis in subjects with potential nonatopic nasal and sinus disease but positive allergy skin tests. The presence of "asymptomatic" allergic rhinitis, and subjects with incidentally positive skin tests requires further investigation [10,29,30]. Allergy skin tests may not be the optimal method for assessing Type I hypersensitivity and other immune responses to fungi [31].

In one approach, we have used a Rhinitis Score to assess symptom severity [32,33]. A predefined threshold defined a positive Rhinitis Score [11]. When matched with skin test results in a 2 × 2 table, we defined those with positive skin tests and Rhinitis Scores as "allergic rhinitis", positive Rhinitis Scores with negative skin tests as "nonallergic rhinitis", negative Rhinitis Scores but positive skin tests as "potential atopy" (asymptomatic allergic rhinitis?), and negative Rhinitis Scores and skin tests as "non-rhinitis" subjects. Shortcomings included: (i) the vagaries of retrospective symptom reviews; (ii) patient preconceptions of "allergy" and "sinus" problems; (iii) difficulty in correlating the timing of symptoms with pollen, dander and mite allergen triggers; (iv) long-term severity assessments in seasonal as opposed to perennial allergic or nonallergic rhinitis; (v) relatively milder symptom scores by younger subjects even when active allergic rhinitis was present; (vi) the need for nasal allergen provocation tests to confirm the diagnosis of allergic rhinitis in borderline allergen skin test positive or negative subjects [34]; and (vii) and the absence of an independent, objective indicator of nasal inflammation such as eosinophilia by nasal scrapings or allergen-specific IgE in nasal secretions.

Allergic disease may be over diagnosed if only a single positive allergy skin test or radioimmunoadsorbant test result was used as the threshold criterion. Two positive skin tests to geographically relevant seasonal or year-round aeroallergens that correlated with typical allergic symptoms represented our minimum criteria [11]. The rate of positive allergy tests in the general population has been widely reported in studies of the prevalence of atopy in CRS. These factors make it difficult to infer causality between the two common, but potentially independent disorders of atopy and polyposis. This difficulty has been compounded in clinical studies by lumping all CRS subjects together. The far right column of table 7 illustrates this effect. These cumulative data obscure the results from specific individual variables (e.g. aspirin sensitivity) best discriminate between the phenotypic categories of CRS.

**Table 7:** Proposed algorithm for the classification of chronic rhinosinusitis. The numbers of subjects in each category and for each variable were extrapolated to a sample size of 100 based on the current data. The 4 categories were generated from the 3^rd ^Decision. The numbers of projected subjects per category (and % per category) were shown in each column. The far right column gives the sum for each variable per 100 CRS subjects.

**Chronic Rhinosinusitis (CRS; n = 100)**					
1^st ^Decision: Polyps	CRS with Nasal Polyps		CRS without Nasal Polyps		N
	
	**Present: N = 50.5**		**Absent: N = 49.4**		50.5

2^nd ^Decision: Aspirin sensitivity	Nasal Polyps with Aspirin Sensitivity (NPasa)	Nasal Polyps with Other Features (NPother)	CRS without (s) Nasal Polyps (CRSsNP)	Osteomeatal Complex Disease (OMC)	
	17.1 (airways)	0	3.6 (urticaria)	4.8 (urticaria)	25.5
3^rd ^Decision: Sinus mucosal thickening	17.1	33.3	28.3	21.2 (normal sinuses)	78.7
4^th ^Decision: FEV_1_/FVC<70% FEF_25%–75%_<70%	12.8 (75%) 15.6 (91%)	13.7 (41%) 18.3 (55%)	5.9 (21%) 8.2 (29%)	3.0 (14%) 9.1 (43%)	35.4 51.2
5^th ^Decision: Peripheral eosinophils >4% Eos. + asthma: a. atopic b. nonatopic c. Eos/no asthma	11.1 (65%) 3.0 (18%) 8.0 (47%) 0.0 (0%)	13.1 (39%) 6.9 (21%) 3.0 (9%) 3.0 (9%)	9.4 (33%) 5.9 (21%) 2.0 (7%) 1.0 (4%)	4.0 (19%) 0 (0%) 2.0 (9%) 2.0 (9%)	37.6 15.8 15.0 6.0
6^th ^Decision: IgE < 10 IU/ml Low IgE + low IgG1 or IgG3	4.9 (29%) 2.4 (14%)	5.5 (17%) 1.4 (4%)	12.5 (44%) 9.0 (32%)	9.5 (45%) 2.1 (10%)	32.4 14.9
7^th ^Decision: Positive allergy skin tests a. seasonal only b. persistent c. negative d. Excess atopy cases per group	7.5 (44%) 1.0 (6%) 6.5 (38%) 9.6 (56%) -0.3 (-2%)	23.6 (71%) 1.0 (3%) 22.6 (68%) 9.7 (29%) 7.9 (24%)	19.2 (68%) 2.0 (7%) 17.3 (61%) 9.1 (32%) 7.1 (25%)	8.7 (41%) 0 (0%) 8.7 (41%) 12.5 (59%) -2.0 (-9%)	59.0 4.0 55.1 40.9 12.7

Total per Group	17.1 (100%)	33.3 (100%)	28.3 (100%)	21.2 (100%)	99.9

Eosinophilia was a common finding in CRS, but again was most frequently associated with aspirin sensitive polyposis (NPasa). Syndromes such as NARES and BENARES may be precursor states for CRS with nasal polyposis [6]. IL-5 is a powerful eosinophilopoeitic factor, and elevated tissue levels may predict a poor prognosis after surgery [35]. Release of local eosinophil chemotactic and survival factors may initiate a self-sustaining eosinophilic inflammatory state independent of Th2 or other lymphocytes [36]. This hypothesis challenges the potential pathological link between eosinophilic allergic rhinitis and eosinophilic CRS [37,38]. Similarities in the tissue cytokine profiles between eosinophilic (allergic and not allergic) and neutrophilic nasal polyps (as in cystic fibrosis) raise additional doubts about the role of atopic mechanisms in CRS [38,39]. Other CRS classification systems have reached a similar conclusion. Kountakis et al. proposed that CRS be stratified in a 2 × 2 factorial manner by the presence or absence of polyps and eosinophilic vs. noneosinophilic (neutrophilic) histopathology [39]. A potential confounding factor may be the preoperative use of oral glucocorticoids to reduce mucosal inflammation and eosinophilia [40]. However, significant differences were noted despite this treatment. CRS with eosinophilia, neuropathy, granulomas, and other findings may suggest Churg-Straus syndrome, Wegener's granulomatosis, and other rare systemic disorders [41].

These findings make it clear that strict subject characterization with data stratification will be imperative for future investigations into mechanisms of CRS.

This conclusion was reinforced by the discovery that nonpolypoid ("hyperplastic") thickening of the mucosa may represent glandular hypertrophy [7,8,42,43]. Those with polypoid changes had destruction of the normal mucosal architecture even in the early stages before gross polyps were identified [7,8,24]. This replacement of normal mucosal glands, nerves, and venous sinusoids by the expanding "edematous sac" would have definite detrimental effects on normal nasal functions such as humidification, glandular exocytosis of host defense proteins, and normal nasal airflow. The "hypertrophic", nonpolypoid subject group was found to have relatively normal mucosal structures except for greatly enlarged areas devoted to submucosal glands. The percent area of Alcian Blue-staining mucous cells was significantly higher in the glandular hypertrophy than the polypoid subjects [8]. Additional radiological, histological, and mRNA microarray data support Malekzadeh's hypothesis of polypoid and glandular hypertrophic forms of CRS [6,7,24,42-47]. Inclusion of these two distinct histopathological subtypes within a single, monolithic category of CRS may be a major cause for the controversies surrounding the pathology, diagnosis and treatment algorithms developed for CRS. "Lumping" of all results into a single disease entity would also explain the difficulty in developing constructive models when limited sets of CRS data were assessed without stratification [24-26,32,35].

A remarkable finding in the nonpolypoid CRS subjects was the high frequency of reduced IgE levels (< 10 IU/ml) and IgG subclass deficiencies. The association of CRS with humoral deficiencies of IgA, IgG subclasses, and all immunoglobulins (e.g. common variable hypogammaglobulinemia, Bruton's agammaglobulinemia) has long been recognized [20,22,48,49]. Precise mechanisms leading to these low antibody levels may include dysfunctional antigen presentation, T cell help, B cell heavy chain switching or other potential mechanisms [50]. Inactivation of these systems may induce compensatory but inappropriate or ineffective immune mechanisms. Overactivity of inappropriately triggered, poorly regulated, or effusive immune responses may contribute to some forms of CRS [51]. Distinct patterns of cytokine mRNAs and cellular protein production in different CRS phenotypes support this contention [52,53].

The nonpolypoid group was divided into those with significant sinus disease by CT scan (May Grades 2, 3 or 4), and those with mild disease limited to the osteomeatal complex (OMC, May Grade 1). The CRSsNP group had higher frequencies of immunoglobulin deficits and positive allergy skin tests. We propose that this group had a reduced capacity to sterilize their sinuses due to dysfunctional humoral immune mechanisms, and that they developed alternative, overcompensating, but inappropriate, chronic immune responses. Multiple mediator pathways [54] may have led to a final common pathway of glandular hypertrophy with mucosal thickening and increased mucus production. The immune deficits may have been progressive, since several of these subjects have progressed to common variable hypogammaglobulinemia (reduced levels of all seven isotypes).

OMC may represent a hybrid group that could progress to polypoid or glandular hypertrophy pathologies, develop allergic rhinitis alone, or regress. It would be necessary to perform middle turbinate biopsies with longitudinal follow-up to answer this question of disease progression. These subjects represent a legitimate category of CRS [55-60]. However, some of these subjects may have had incidental alterations or false positive CT scans. Incidental abnormalities including asymptomatic pansinusitis have been noted in the common cold and up to 32% of subjects having CT or MRI scans to assess headache, orbital, and intracranial disease [61-64]. Nasal blockage was the only questionnaire item to be significantly associated with abnormal scans [65]. "Blockage" has been associated with persistent allergic rhinitis, while "sneezing and running" was more typical in intermittent allergic rhinitis where histaminergic mechanisms may predominate [66]. These findings illustrate the need to use multiple, rigorously defined historical, physical examination, questionnaire, radiological, and other criteria for evaluating CRS.

A final group of subjects have been separately identified [58]. They have severe, continued nasal, sinus and facial complaints suggestive of CRS [11], sinus region tenderness (regional hyperalgesia) [16], minimal sinus disease by CT scan (JNB, personal observation), and mucosal secretory dysfunction [18] despite surgery, antibiotics and other standard treatments. We have proposed that this group may be a component of the chronic fatigue syndrome spectrum of illnesses. Chronic fatigue syndrome criteria were met by 26% of this CRS population. This was much higher than in the general population (estimated 2%) [67]. Factor analysis of the CRSsNP group excluded chronic fatigue syndrome subjects since they had a negative loading factor. This provided evidence that this syndrome was not related to mucosal hypertrophy, humoral immunity, or atopy. Instead, these subjects may have dysfunctional spinal dorsal horn and central nervous system regulation of pain (systemic hyperalgesia), autonomic instability, limbic, anterior cingulate, amygdala and other cortical disruptions. [68]. These changes contribute to defective emotional, memory and executive decision making processes. Neural dysfunction may augment the magnitude of sinus region hyperalgesia and allodynia complaints, parasympathetic reflex-mediated glandular secretion, and responses to nociceptive nasal provocations in chronic fatigue syndeom [18]. However, the pathogenesis and mechanisms of regional and systemic hyperalgesia in chronic fatigue syndrome were unlikely to be related to CRS, since CT scan severity scores and pain symptoms were not correlated in CRS [69]. Rhinitis and sinusitis complaints in these syndromes likely represent irritant rhinitis [15] that must be discriminated from allergic and CRS disease.

### Diagnostic algorithm

The stratification and factor analyses were used to develop an algorithm for the evaluation of CRS, and to predict the provisional distributions of CRS subjects using this classification scheme (table 7). The order of decisions was based on the frequency of each variable and their ability to define subgroups based on potential pathogenic mechanisms.

The 1^st ^decision regarded polyps. Their presence or absence divided the CRS group in half.

The 2^nd ^decision was whether there was a strong history or evidence from provocation testing of aspirin or other nonsteroidal anti-inflammatory drug sensitivity with airway obstruction. Positive subjects represented the NPasa category. Other causes must have predominated in the remainder of polyp subjects (i.e. NPother). Angioedema did not discriminate between groups.

The 3^rd ^decision was based on a CT scan that showed sinus mucosal thickening > 5 mm or more extensive and severe abnormalities (May Classes 2, 3 and 4). This extent of disease in the absence of polyps defined the CRSsNP group. The OMC group (May Class 1) was limited to nasal disease. These three decisions defined our four major categories.

The 4^th ^decision was based on pulmonary function. The NPasa group had significantly worse airway function that the other groups. The qualitative reduction of FEV_1_/FVC ratio to below 0.70 was the single most significant discriminating variable. Small airways function was present in 91% of NPasa, and near 50% in the other three groups (table 2). Independent polypoid and atopic mechanisms may contribute to the links between CRS and asthma in the 4 categories.

The 5^th ^decision was to determine if the peripheral blood eosinophil count was > 4%. Other thresholds such as absolute cell counts may be more sensitive, but were not examined here. Eosinophilia was associated with asthma and negative skin tests in 47% of the NPasa group. By contrast, 21% of the NPother and CRSsNP groups had eosinophilia and asthma with positive skin tests (atopic asthma). Tissue eosinophilia may have been an even more discriminating marker [39].

The 6^th ^decision was immune status. IgE was < 10 IU/ml in 44% of the nonpolypoid CRS subgroup, compared to 21% when polyps were present (p = 0.015 by Fisher's Exact test). The small number of subjects per group meant that statistical significance was lost when each CRS group was compared. Groups of at least 30 subjects each should facilitate investigation of this finding. The combination of low IgE plus either low IgG1 or IgG3 was more prevalent in the CRSsNP group.

The 7^th ^decision was based on skin test reactivity (table 3 and factor analysis). Positive skin tests to the persistent allergens (dust mites, cat, and fungi) dominated with only 4% of CRS subjects having solely seasonal patterns (trees, grasses, weeds). Based on the extrapolations of table 7, the allergic contingents within the NPother and CRSsNP categories accounted for 43% of the CRS population, and contributed 12.7 excess cases of atopy per 100 subjects compared to the normal reference population. The majority were sensitized to dust mites.

The low rating for this 7^th ^decision should be cautionary. The NPother and CRSsNP groups were almost identical in their patterns of allergic sensitization. However, these similarities do not explain why half develop polyps, while the other half do not. It may be that the mucosal microenvironment promotes Th2 reactivity and atopic sensitization to persistent allergens. However, other environmental, genetic, and molecular influences that remain to be discovered may force the inflammatory cascade to diverge into the mutually exclusive polypoid and glandular hypertrophy histological subtypes of CRS [7,8]. The perplexing prevalence of low IgE in the otherwise highly allergic CRSsNP group was distinctly different from the NPother group. It suggests that immune dysregulation may contribute to CRSsNP pathophysiology.

## Conclusion

This retrospective analysis provides justification for the consensus division of CRS into groups with and without nasal polyps. Information about aspirin sensitivity, clinical asthma, airflow obstruction, the extent of the sinusitis disease process, immunoglobulin isotype deficiencies, and allergy skin test results provided logical criteria for the subdivision of these two groups. Polyposis subjects were subdivided based on the presence (NPasa) or absence of historical aspirin sensitivity (NPother). NPother subjects were further subdivided by factor analysis into subsets with (2/3) and without (1/3) allergy. The non-polypoid subjects were divided based on CT scan severity. The CRSsNP group had sinus mucosal thickening (May Class 2, 3, or 4), reduced immunoglobulin levels, and allergy. Those with disease limited to the nasal osteomeatal complex for the last group (OMC). Immunoglobulin deficiencies and atopy were present to some extent in all these groups, but were most significantly associated with nonpolypoid disease. These distinctions also have relevance to asthma studies, where atopic (n = 500/700) and nonatopic (n = 200/700) asthmatics can be clinically distinguished [70]. The current results provide a logical framework for stratification of CRS subjects for future studies of disease diagnosis, treatment and pathogenic mechanisms.

## Abbreviations

CRS, chronic rhinosinusitis; NPasa, **n**asal **p**olyps with **aspirin **sensitivity; NPother, polypoid CRS in the absence of aspirin sensitivity; CRSsNP, nonpolypoid CRS with sinus mucosal involvement by CT scan ("s"=without); OMC, nonpolypoid CRS with **o**steo**m**eatal **c**omplex narrowing on CT scan; NARES, nonallergic rhinitis with eosinophilia syndrome; BENARES, blood eosinophilia with NARES.

## Competing interests

The author(s) declare that they have no competing interests.

## Authors' contributions

JNB conducted the clinical project. HM supervised the confidential and anonymous entry of data, and its statistical review. The manuscript was written jointly by the two authors.
